# Combined MRI morphometry and source imaging guide placement of stereo-EEG electrodes in focal epilepsy with subtle or absent lesions

**DOI:** 10.3389/fneur.2025.1685431

**Published:** 2025-11-18

**Authors:** Peter C. Reinacher, Dirk-Matthias Altenmüller, Julia M. Nakagawa, Yiwen Li Hegner, Cristian Dorin Antal, Matthias Dümpelmann, Theo Demerath, Anke M. Staack, Hans-Jürgen Huppertz, Soroush Doostkam, Horst Urbach, Andreas Schulze-Bonhage, Marcel Heers

**Affiliations:** 1 Department of Stereotactic and Functional Neurosurgery, Faculty of Medicine, Medical Center—University of Freiburg, Freiburg, Germany; 2 Fraunhofer Institute for Laser Technology (ILT), Aachen, Germany; 3 Epilepsy Center, Faculty of Medicine, Medical Center—University of Freiburg, Freiburg, Germany; 4 Department of Neurosurgery, Faculty of Medicine, Medical Center—University of Freiburg, Freiburg, Germany; 5 Translational Epilepsy Research, Department of Neurosurgery, Faculty of Medicine, Medical Center -University of Freiburg, Freiburg, Germany; 6 Department of Neurology and Epileptology, Hertie Institute for Clinical Brain Research, University of Tübingen, Tübingen, Germany; 7 Epilepsy Unit, Alfred Hospital, Melbourne, VIC, Australia; 8 University of Medicine and Pharmacy "Grigore T. Popa", Iasi, Romania; 9 Department of Neuroradiology, Faculty of Medicine, Medical Center—University of Freiburg, Freiburg, Germany; 10 Epilepsy Center Kork, Kehl-Kork, Germany; 11 Swiss Epilepsy Center, Klinik Lengg, Zürich, Switzerland; 12 Institute of Neuropathology, Faculty of Medicine, Medical Center—University of Freiburg, Freiburg, Germany

**Keywords:** focal epilepsy, MRI morphometry, electric source imaging, magnetic source imaging, stereo-EEG, presurgical epilepsy diagnostics

## Abstract

**Introduction:**

Planning stereo-electroencephalography (sEEG) for focal drug-resistant epilepsy with subtle or absent lesions requires accurate non-invasive spatial information about the hypothetical organization of the epileptic focus. The targeting of individual trajectories for a limited number of invasive depth electrodes is particularly challenging in patients who have undergone prior epilepsy surgery. This study investigated how information from multimodal imaging can guide sEEG planning and enable successful epilepsy surgery in patients with non-lesional focal epilepsy.

**Methods:**

We studied 15 patients who appeared non-lesional on conventional MRI and were suspected to have mono-focal epilepsy. These patients underwent sEEG implantation between October 2019 and October 2022, based on findings from non-invasive video-EEG monitoring and multimodal imaging. Among the participants, four had undergone prior epilepsy surgery, including three who had previously undergone invasive EEG. All patients underwent high-resolution 3 T MRI and MRI morphometry (MAP) as part of their non-invasive presurgical diagnostics. Electric and magnetic source imaging were performed in patient subgroups. sEEG planning incorporated findings from the available imaging methods registered within the stereotactic planning system.

**Results:**

A median of nine sEEG electrodes (range: 7–11) were implanted in each patient, targeting both primary and secondary hypotheses about the epileptic focus location. sEEG recordings revealed a monofocal seizure onset in 12 out of 15 patients, all of whom subsequently underwent epilepsy surgery. No bleeding complications occurred. Of these patients, nine achieved Engel 1 postsurgical outcomes, while three had Engel ≥2 outcomes. Surgery was not performed in three patients due to multifocal epilepsy (n = 2) or an unidentified seizure onset zone (SOZ, n = 1). Concordance across multiple imaging modalities was associated with favorable surgical outcomes.

**Conclusion:**

In patients with focal epilepsy and subtle or absent lesions, sophisticated sEEG diagnostics guided by advanced multimodal imaging can successfully identify the seizure onset zone. When focal onset is confirmed and multifocal epilepsy is excluded through sEEG, subsequent epilepsy surgery often results in seizure-free outcomes.

## Introduction

Epilepsy surgery is the treatment of choice for drug-resistant focal epilepsy (1). Successful surgery significantly improves quality of life in patients with focal epilepsy (2). Multiple parameters influence the success of epilepsy surgery in stopping seizures. Outcomes are typically less favorable in patients with non-lesional focal epilepsy compared to patients with focal lesional epilepsy (3–5). However, non-lesional focal epilepsy accounts for up to 50% of all focal epilepsy patients (6). Previous epilepsy surgery can decrease the chance of a favorable postsurgical outcome (7), and extratemporal lobe epilepsy is considered more difficult to treat with epilepsy surgery compared to temporal lobe epilepsy. Still, the success rates for treating extratemporal lobe epilepsy have improved over time (8).

Especially in non-lesional focal epilepsy and focal epilepsy where previous epilepsy surgery has failed, stereo-EEG (sEEG) serves as an invasive diagnostic procedure. This procedure enables epilepsy surgery in a group of patients with non-invasive electro-clinical findings that confine the hypotheses about the seizure onset zone (SOZ) to only a few potential localizations. In general, the likelihood of forming clear hypotheses about the SOZ without structural lesions is extremely low, and the opportunity for epilepsy surgery after sEEG is limited in non-lesional epilepsy cases (9). The main drawback of sEEG is its ‘tunnel view,’ as it only records cortical brain activity from local brain tissue with a diameter of approximately 1 cm (10). However, epilepsy surgery after sEEG with electrodes placed in key positions to reliably delimit the epileptic focus can lead to postsurgical outcomes that are not inferior to those of patients with lesional focal epilepsy (11).

**Figure 1:**
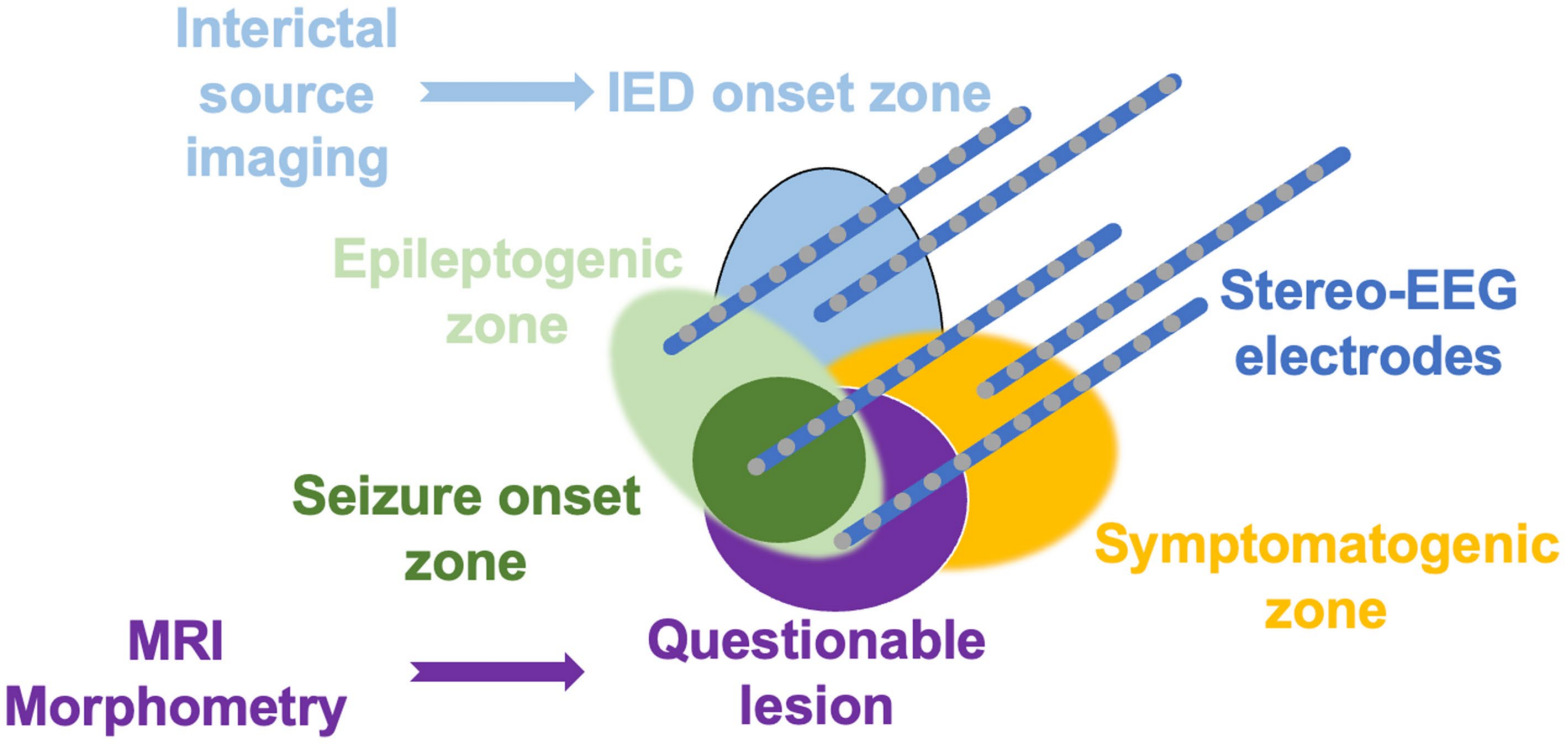
Based on the traditional model of the organization of the epileptic focus (12), the figure highlights the contribution of multimodal imaging in guiding the placement of sEEG electrodes in patients with non-lesional focal epilepsy. While MRI morphometry enhances the detection of subtle structural lesions, interictal source imaging delimits the irritative zone to non-invasively identify the interictal epileptiform discharge (IED) onset zone.

Recent technical advances in neuroimaging have made multimodal imaging available to guide the planning of sEEG electrode implantation for non-lesional focal epilepsy patients. Once clear electro-clinical hypotheses about the epileptic focus are defined (12, 13), different zones can be targeted for further exploration based on findings from multimodal imaging (Figure 1). Individual sEEG electrode placement will benefit from several imaging modalities with diverse spatial–temporal information provided by each imaging method. All these different methods must be accurately co-registered to apply advanced strategies for the effective placement of sEEG electrodes (14).

High-resolution 3 T MRI is a key element in presurgical epilepsy diagnostics (15). Integrating MRI morphometry with traditional visual MRI analysis has become a relevant tool for detecting subtle MRI abnormalities, particularly in identifying focal cortical dysplasias (FCDs) or mild malformations of cortical development (16–19). However, MRI morphometry often detects false-positive clusters. Furthermore, determining the spatial extent of FCDs remains challenging, even with advanced MRI morphometry protocols; therefore, sEEG is often warranted (16, 20). For multimodal image co-registration, high-resolution isotropic MRI sequences serve as the backbone. These sequences are used for image co-registration to create patient-specific head models and perform source imaging (21).

Several prospective trials have demonstrated that magnetic and electric source imaging (MSI and ESI) can lead to clinically significant changes in the placement of intracranial EEG electrodes (22, 23). ESI from high-density EEG (hdEEG) with more than 64 EEG electrodes (24) and ESI from long-term monitoring (LTM-ESI) provide evidence of their contribution to presurgical epilepsy diagnostics and guidance of sEEG electrode placement (25–27). The high temporal resolution of both ESI and MSI allows the identification of the IED onset zone (28–30).

To date, there have been very few studies that included multimodal imaging co-registration and validation in sEEG planning. A typical pipeline for presurgical planning often only comprises regions of interest created from MRI morphometry (31, 32), without considering findings from other methods.

### Aims

This study examines the spectrum of focal epilepsy patients in whom conventional MRI does not reveal clear lesions—ranging from truly non-lesional patients to those with subtle structural abnormalities detectable only through advanced imaging techniques. We hypothesize that taking advantage of the added information from different available imaging modalities leads to optimized strategical placement of sEEG electrodes and subsequent successful epilepsy surgery. Multimodal imaging may enable the demarcation of the SOZ through invasive diagnostics using a minimum number of sEEG electrodes as the basis for successful epilepsy surgery.

## Methods

### Patients

Between October 2019 and October 2022, we retrospectively included all 15 patients (9 female individuals, age at onset 11.8 years (± 6.4 years)) who appeared non-lesional on conventional MRI evaluation and underwent sEEG for focal epilepsy at the Epilepsy Center, University Hospital Freiburg. The mean epilepsy duration was 19.8 years (± 8.8 years; details in Table 1).

**Table 1:** Concordance of multimodal imaging methods.

ID	Age onset (y)	Duration (y)	Semiology lateralization correct	ASM	SOZ/resection	MAP	hdEEG	LTM	MSI	Prior surgery/invasive EEG	Histology	Outcome (follow-up)
1	4	25	Yes	BRV, OXC	RT pole	**C**	**C**	NA	NA	RF	mMCD	1 (12)
2	15	15	Yes	BRV, ZNS	LF	neg	NA	NA	NA	no	NA	NS
3	12	12	Yes	CBZ, LEV	LCe	**C**	**C**	**C**	NA	LF pole/subdural	FCD II	1 (14)
4	13	10	Yes	BRV, OXC	RTOb	**C**	**C**	NA	NA	no	Gliosis	1 (26)
5	18	2	Yes	BRV, LTG	LPoperc	**C**	nC	nC	**C**	no	FCD II	1 (24)
6	2	18	Yes	LTG, LEV	RForb	**C**	**C**	NA	**C**	no	mMCD	1 (18)
7	29	4	Yes	BRV, LCM	LFlat	**C**	NA	NA	NA	no	FCD II	1 (18)
8	13	30	No lateralization	BRV, CBZ	multifocal: bil lateral, mesial, basal TO	**C**	NA	NA	NA	no	NA	NS
9	15	19	Yes	LTG, LEV, PGB	RTpole, RAH	neg	NA	NA	NA	no	mMCD	1 (21)
10	7	25	Yes	LTG	RF, lat sup	neg	neg	**C**	neg	no	mMCD	2 (14)
11	14	22	Yes	CNB, LTG	LF, lat sup	neg	neg	NA	neg	no	FCD II	1 (14)
12	13	20	Yes	CNB, LEV	LTO mes/lat multifocal	**C**	nC	NA	NA	no	NA	NS
13	11	34	Yes	LTG, BRV, PER	RF, lat inf (prec)	neg	nC	NA	NA	RF operc/Stereo-EEG	Gliosis	3 (18)
14	6	21	Yes	LTG, PER	LT post	neg	NA	NA	NA	RT pole	Gliosis	4 (14)
15	5	39	No lateralization	BRV, LCM, PGB	LForb	nC*	nC*	NA	NA	LT pole/subdural	FCD II	1* (12)

A: amygdala, b: basal, bil: bilateral, C: concordant, Ce: central, F: frontal, FCD II: focal cortical dysplasia type II, H: hippocampus, inf: inferior, L: left, lat: lateral, mes: mesial, mMCD: mild malformation of cortical developement, NA: not available, nC: not concordant, NS: no surgery, R: right, sup: superior, T: temporal, O: occipital, operc: opercular, orb: orbital, post: posterior, prec: precentral, y: years, ASM: anti-seizure medication, BRV: brivaracetam, CBZ: carbamazepine, CNB: cenobamate, LCM: lacosamide, LEV: levetiracetame, LTG: lamotrigine, OXC: oxcarbazepine, PGB: pregabaline PER: perampanel, *secondary cluster concordant refers to MRI morphometry clusters initially not visually confirmed on fluid-attenuated inversion recovery (FLAIR) MRI or counts of interictal epileptiform discharges (IEDs) lower than the primary IED cluster in ESI from high-density EEG (hdEEG-ESI). Outcome: Postsurgical results according to the Engel classification (follow-up in months). The Bold values emphasize the “C” for “concordant”.

### Non-invasive and invasive long-term video-EEG monitoring

All included patients underwent non-invasive video-EEG monitoring either at the Epilepsy Center, Freiburg, Germany, or at the Epilepsy Center Kork, Kehl-Kork, Germany. All subsequent invasive sEEG investigations were performed at the Epilepsy Center, Freiburg, Germany. sEEG data were acquired using Nuevo telemetry systems (Compumedics, Abbotsford, Victoria, Australia). sEEG electrodes with a contact length of 2 mm and an intercontact distance of 2.5 mm (AD-Tech, Oak Creek, WI, USA) were used. During monitoring, anti-seizure medication (ASM) was tapered to provoke seizures.

### 3T MRI and MRI morphometry (MAP)

All patients included in this study underwent a 3 T Prisma MRI of the brain (Siemens Healthcare, Erlangen, Germany) using a dedicated epilepsy protocol described elsewhere (15). The protocol includes 3D magnetization-prepared rapid acquisition gradient echo with T1 weighting (T1 MPRAGE), fluid-attenuated inversion recovery (FLAIR), and magnetization-prepared 2 rapid acquisition gradient echo (MP2RAGE) sequences with 1 mm isotropic voxels. All patients underwent MP2RAGE-based MRI morphometry using the Morphometric Analysis Program (MAP) v2018 with a scanner-specific normative dataset (16, 33). Since false-positive clusters can appear in morphometric maps, the findings were reviewed by two neuroradiologists (H. U. and T. D.) and visually compared with findings from the original MR images, particularly the FLAIR sequence (resolution 1 mm, isotropic voxels). The visual review yielded one or no MRI morphometry cluster per patient.

### ESI from high-density EEG (hdEEG-ESI)

A subset of 10 patients underwent high-density EEG with 256 scalp EEG electrodes (MagStimEGI, Eugene, OR, USA). The recording duration was between 1 and 1.5 h without any further provocation methods. For ESI based on hdEEG, IEDs were marked visually and averaged by type. If multiple IED types per patient were detected, only the most frequent IED type per patient was considered for the quantitative comparison of this project. We decided to focus on the most frequent IED type per patient as it most likely spatially co-localizes with the SOZ (29, 34). A three-compartment boundary element head model (BEM) was created from each patient’s individual T1-weighted MPRAGE MRI. MRI and hdEEG electrode positions were coregistered using label matching. ESI was conducted using the inverse method sLORETA (35) within Curry 9 software (Compumedics Neuroscan, Hamburg, Germany).

### Semi-automated ESI from LTM (LTM-ESI)

ESI from LTM was conducted in a subset of three patients who underwent scalp EEG LTM using 40 evenly spaced EEG electrodes from the 10/10 and 10/5 systems, including AF11 and AF12, as described elsewhere (34). IEDs were clustered into IED types using Persyst 14c, Persyst Inc., Solana Beach, CA, USA. ESI was performed on the average IEDs of the most frequent IED type per patient using Curry 9 software. Only IEDs with a similarity index of > 0.5 were selected. For the same reasons as in the hdEEG-ESI analysis, we focused our quantitative analysis on the most frequent IED type per patient. Within Curry 9, a three-compartment BEM was created from each patient’s individual 3D T1-weighted MPRAGE MRI. ESI was conducted using the sLORETA algorithm (35, 36).

### MSI from simultaneous MEG/EEG

In a subset of four patients, simultaneous magnetoencephalography (MEG) and EEG were recorded at the MEG Center, University Hospital Tübingen, Tübingen, Germany, using a 275-channel MEG system (CTF, Coquitlam, BC, CA) and a 31-channel EEG cap (Easycap, Etterschlag, Germany). Electrode positions and the head shape were identified using a Polhemus system (Polhemus, Colchester, VT, USA). For MSI, the patient’s MRI was coregistered with the MEG data based on landmarks and refined by head shape and electrode positions. A three-compartment BEM was created within Curry 9 software from the patient’s individual T1-weighted MPRAGE MRI. Fusion of MEG and EEG signals was used for source imaging with sLORETA or MUSIC (35, 36) within Curry 9 software.

### Co-registration of multimodal imaging findings

Findings from MRI morphometry, ESI, MSI, and FDG-PET were visualized using Curry 9 software for the review of multimodal imaging data. For sEEG planning, multimodal data were imported into the Brainlab neuronavigation system (Image Fusion and Distortion Correction Cranial 4.5.0.56, Object Management and SmartBrush 4.5.0.71, Stereotaxy 2.6.0.539, Brainlab, Munich, Germany).

### Evaluation of contribution to invasive sEEG and postsurgical outcomes

Quantifying the exact contribution of individual methods to invasive sEEG planning is challenging. Therefore, an indirect approach was used, based on the concordance of each method’s results with the SOZ identified through sEEG. The results were considered concordant if the Euclidean distance between the border of the MRI morphometry cluster or the peak of the ESI/MSI localization and the sEEG electrode contact(s) that recorded the SOZ was less than 1.5 cm. This threshold was chosen to balance the need for precise sEEG electrode placement with the limited field of view of depth electrode contacts and the spatial resolution constraints of functional imaging methods in a clinical setting. All patients who underwent epilepsy surgery had a postsurgical follow-up of at least 1 year (Details in Table 1).

### Statistics

For statistical evaluation, true positive, false positive, and false negative imaging findings were reported. Since we selected the source imaging findings of the most frequent IED type and the MRI morphometry cluster that was visually verified by the neuroradiologist, we had a single finding for each imaging method per patient. True positives were defined as findings that had a Euclidean distance of < 1.5 cm from the sEEG contact that recorded the SOZ, and false positives were findings that had a distance of ≥ 1.5 cm from the sEEG contact that recorded the SOZ. All findings that localized to a different lobe than the sEEG contact that recorded the SOZ were defined as false positives. The findings were counted as false negatives when the diagnostic test was performed but did not yield a result, i.e., no IEDs were recorded in LTM or hdEEG. Due to methodological limitations, there were no true negatives in our cohort.

To evaluate if multimodal concordance was associated with favorable postsurgical outcomes, we dichotomized the patients into two groups: (1) those with an Engel class I outcome and (2) those with an Engel class > I outcome or in whom no epilepsy surgery could be offered. We compared these two patient groups with two categories of imaging findings: (1) none or a single imaging method concordant with the SOZ and (2) more than one imaging method concordant with the SOZ. Fisher’s exact test was used for statistical analysis.

## Results

### MRI morphometry (MAP)

**Figure 2:**
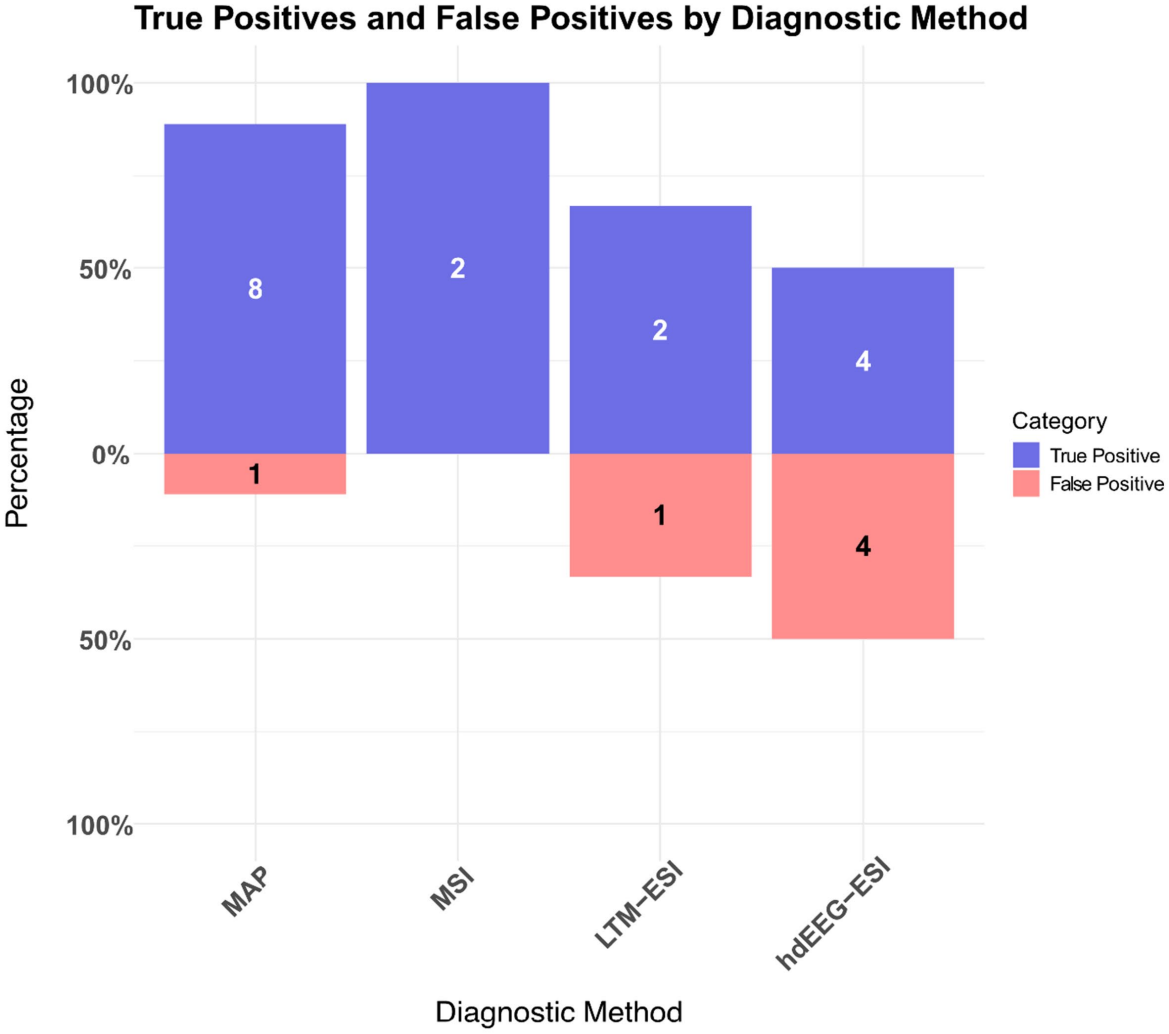
Proportions of true positives (blue) and false positives (red) for each multimodal imaging method, with absolute numbers shown within the bars. MAP showed the highest number of true positives (8/9). MSI and electric source imaging methods showed lower true positive rates, with MSI being concordant in two out of two cases, LTM-ESI concordant in two of three cases and hdEEG-ESI in four of eight cases. Given that each patient had a single finding reported for each imaging method, the resulting counts reflect dimensions at both the imaging method and patient levels. Abbreviations: MAP: MRI morphometry, MSI: MEG/EEG fusion source imaging, LTM-ESI: electric source imaging from long-term video-EEG monitoring, hdEEG-ESI: high-density electroencephalography source imaging. Note that MSI and LTM-ESI were available in fewer patients compared to other methods. A total of six patients had no concordant findings from any imaging modality. The complete results for all patients are detailed in Table 1.

In 8 of 15 patients (53%), a single MRI morphometry cluster verified by visual review from the neuroradiologists was concordant with the SOZ in sEEG (Figure 2). The concordance of MRI morphometry with the SOZ was especially high in patients without prior surgery (Table 1). However, a true positive focal MRI morphometry cluster did not exclude a more extended SOZ (P8, P12). False positive MRI morphometry clusters were often observed, especially in the previously operated patients due to postoperative gliosis; however, these clusters were not confirmed upon visual inspection by the neuroradiologist. In one patient (P15), a visually confirmed morphometry cluster in the left parahippocampal gyrus did not align with the SOZ. Instead, a different cluster in the left orbitofrontal region, which was initially overlooked as a potential FCD, was later identified as relevant.

### hdEEG-ESI

Interictal ESI using hdEEG was conducted in 10 of 15 patients. In 4 out of 10 patients (40%), the ESI cluster with the most frequent IED type was concordant with the SOZ. In 2 out of 10 patients (20%), no IEDs were recorded. In patient 15, the ESI cluster with the second-highest IED count was concordant with the SOZ, but not the ESI cluster with the most frequent IED type. In the statistical comparison, this hdEEG-ESI result was counted as a false negative in this patient because the concordant cluster was not the cluster with the most frequent IED type.

### LTM-ESI

In 3 out of 15 patients, ESI was performed using LTM with a 40-electrode setup, as described earlier. The findings from the cluster with the most frequent IED type were concordant with the SOZ in two out of three patients (67%), while they were not concordant in one out of three patients (33%).

### MSI

MSI was performed in 4 out of 15 patients using simultaneous MEG/EEG recordings. In two patients, the source imaging maximum from MSI was concordant with the SOZ. No IEDs were detected during the recordings in the remaining two patients.

### Combined interpretation of the results

**Figure 3:**
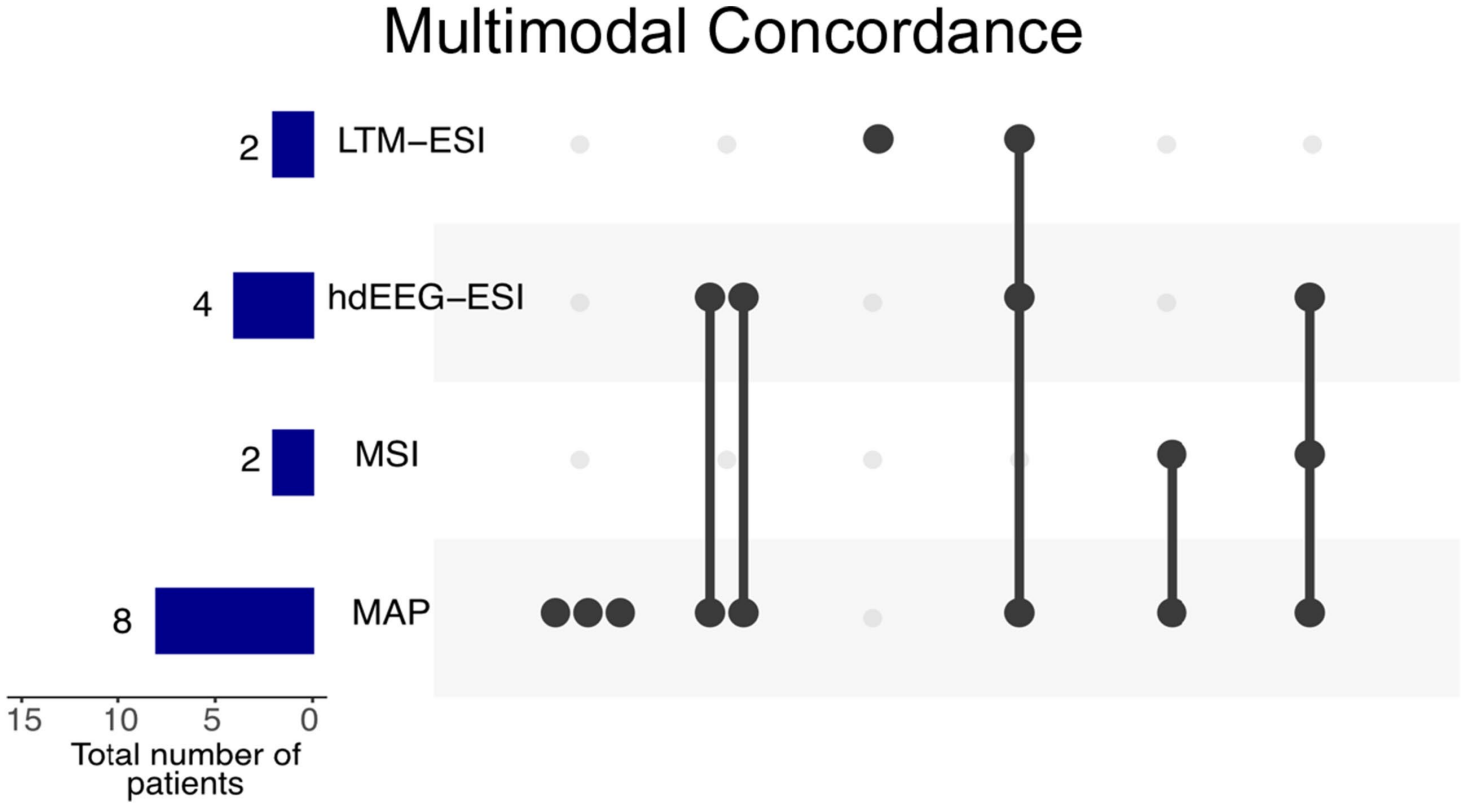
Combinations of diagnostic methods concordant with the seizure onset zone (SOZ). Blue bars on the left indicate the total number of times each method was concordant in the patient cohort, with MAP being the most frequently concordant method (n = 8). Black dots connected with vertical bars represent the number of patients showing concordance for specific combinations of methods. The complete results for all patients, including modality availability and non-concordant findings, are detailed in Table 1. The patients with concordance of multiple imaging modalities (n = 5) had a monofocal SOZ and achieved seizure-free outcomes after surgery. In contrast, the two patients with isolated MAP concordance had a multifocal SOZ and were not considered surgical candidates (P8, P12). Six patients without any concordant findings are not represented in this plot. Modality availability varied across patients: MAP was available in all 15 patients, hdEEG-ESI in 10 patients, MSI in 4 patients, and LTM-ESI in 3 patients.

The interpretation of the combined findings from multimodal imaging, along with the electro-clinical hypothesis of the epileptic focus, guided the targeted placement of sEEG electrodes. While MRI morphometry was the imaging method most frequently concordant with the SOZ, it was supported by one or two functional imaging methods in each patient (Figure 3).

**Figure 4:**
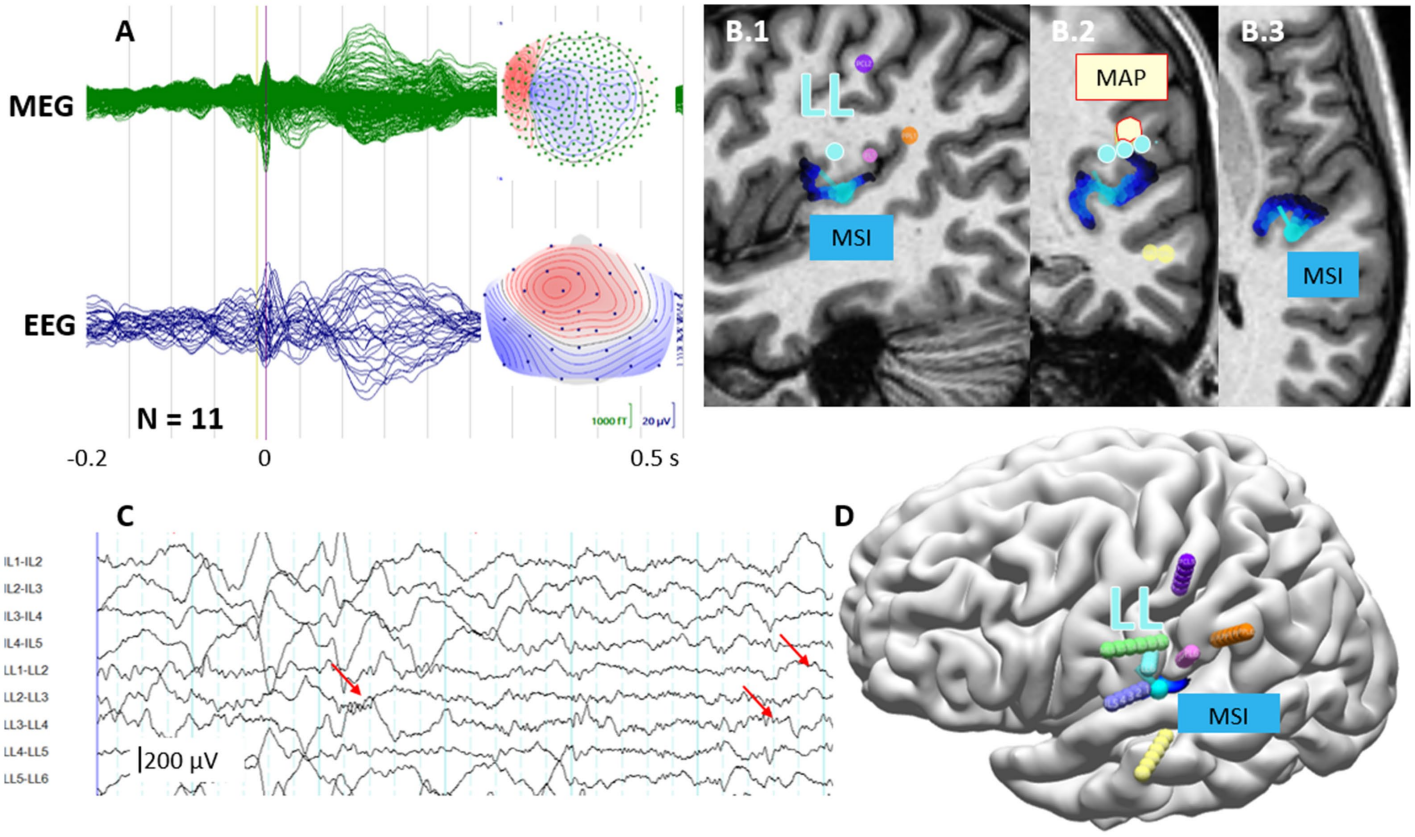
Multimodal concordance analysis in a patient with left parietal lobe epilepsy (P5) demonstrating seizure onset zone localization. **(A)** Averaged interictal epileptiform discharge (IED) butterfly plots from simultaneous MEG (green) and EEG (blue, N = 11 trials), with corresponding field topographies (insets). Topographical maps display negative amplitude maxima (blue) and positive amplitude maxima (red). **(B.1–3)** Multimodal imaging findings overlaid on an MP2RAGE MRI sequence. The morphometric analysis (MAP) cluster, shown in bright yellow, indicates a subtle cortical abnormality. The MEG source imaging (MSI) maximum (dark blue) demonstrates concordance with the seizure onset zone, since it is located <15 mm from electrode contacts LL2-3. **(C)** Stereoelectroencephalography (sEEG) recordings during seizure onset, showing ictal discharge initiation at electrode contacts LL2-3 (red arrows). Electrode label numbers indicate the depth of the electrode contact. **(D)** Three-dimensional rendering of the electrode implantation scheme showing seven invasive sEEG electrodes, with the MSI source maximum (dark blue) in relation to the confirmed seizure onset zone. Color-coded electrodes represent different implantation trajectories targeting the suspected epileptogenic zone.

The combined multimodal approach, leveraging the strengths of each method to test multiple hypotheses about the SOZ, outperformed individual methods applied separately. Advanced imaging methods achieved concordance of at least a single imaging method with the SOZ in 9 out of 15 patients (60%). This underscores the value of integrating multiple diagnostic techniques in the comprehensive identification of the SOZ with sEEG. Concordance among multiple imaging methods was typically observed in the patients with a monofocal SOZ (Table 1; Figures 3, 4).

### sEEG evaluation

After successful sEEG evaluation with the acquisition of habitual seizures, 12 out of 15 patients underwent subsequent epilepsy surgery. In 3 out of 15 patients, no epilepsy surgery could be offered. Two out of 12 patients with sEEG had a multifocal SOZ (P8, P12). Both had unilateral focal MRI morphometry clusters without concordant findings from other imaging modalities. Therefore, MRI morphometry did not reflect the extent of the SOZ in these patients. Only in one patient with frontal lobe epilepsy, the SOZ could not be identified (P2) using sEEG.

### Postsurgical outcomes and histopathology

Concordance of > 1 multimodal imaging method was correlated with favorable postsurgical outcomes (*p* = 0.044, Fisher’s exact test, Table 2). We typically saw concordance of MRI morphometry and an additional method with the SOZ (Figure 3).

**Table 2:** Distribution of the patients by multiple concordant findings and postsurgical outcome.

Multiple concordant Findings	Good outcome (Engel 1)	Poor outcome (Engel > 1/No Surgery)	Total
Yes	5 (100%)	0	5
No	4 (40%)	6 (60%)	10
Total	9 (60%)	6 (40%)	15

Fisher’s exact test: *p* = 0.044; Multiple concordant findings were significantly associated with good outcomes (*p* < 0.05). All patients with multiple concordant findings (*n* = 5) had good outcomes (Engel 1), while only 40% of the patients without multiple concordant findings had good outcomes.

In total, 9 out of 12 patients (75%) who underwent epilepsy surgery became seizure-free. Histopathological evaluation revealed FCD type II in five patients, mMCD in four patients, and gliosis in three patients. Gliosis was found in two patients with unfavorable postsurgical outcomes (P13, P14) and one other patient with seizure-free outcomes (P4).

Among the patients who had previously undergone epilepsy surgery, two patients (P1, P3) achieved a favorable postsurgical outcome when MRI morphometry findings were concordant with ESI from hdEEG. In contrast, a negative MRI morphometry result combined with non-concordant hdEEG-ESI (P13) was associated with an unfavorable postsurgical outcome in this group of patients.

**Figure 5:**
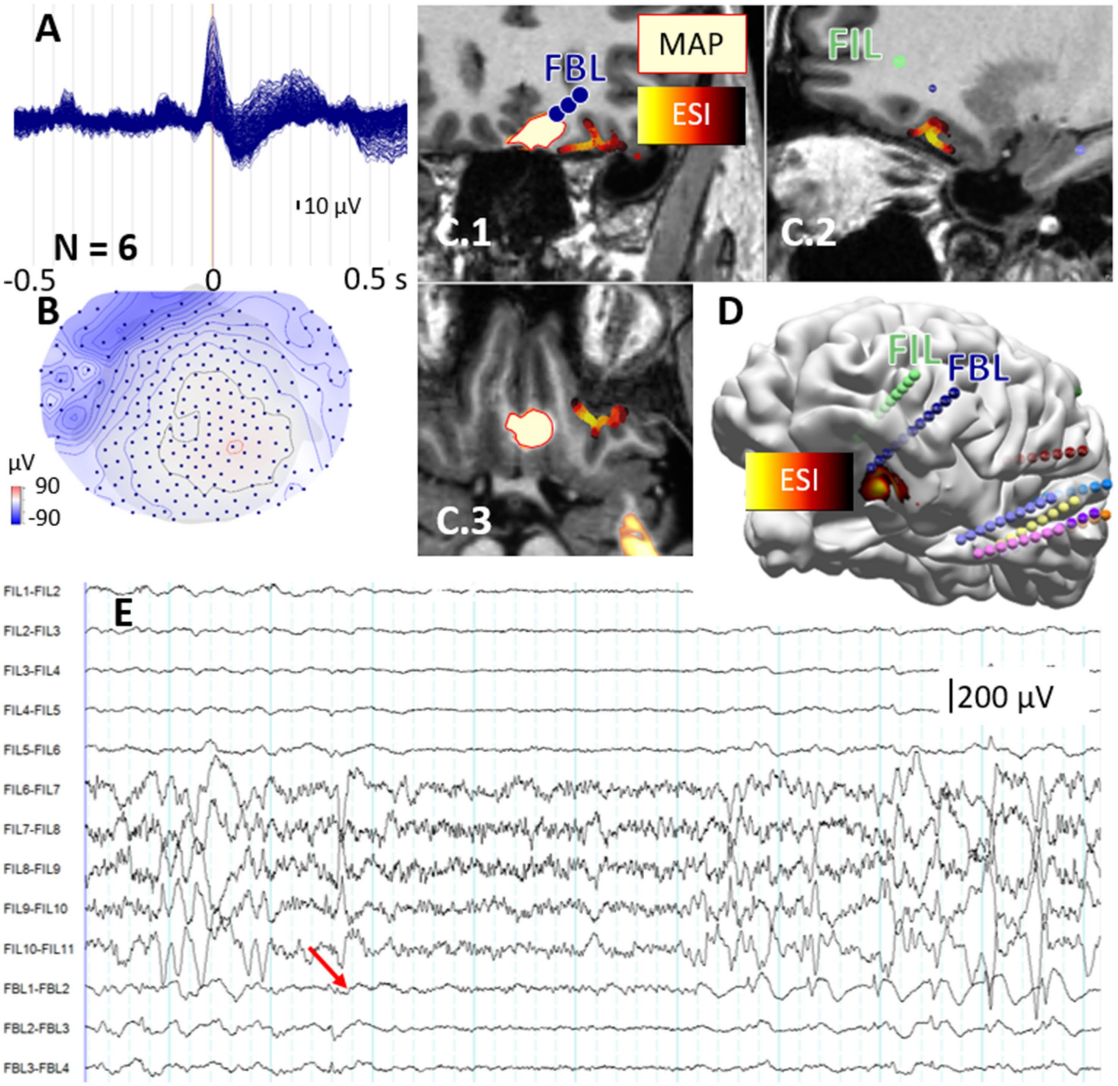
Multimodal imaging concordance in left frontal lobe epilepsy (P15), demonstrating the clinical value of secondary imaging findings. **(A)** Averaged interictal epileptiform discharge (IED) butterfly plot from high-density EEG (hdEEG, N = 6 epochs). **(B)** Scalp voltage topography at the IED peak, displaying amplitude distribution with maximum negativity over left frontal electrodes (blue dots indicate electrode positions). **(C.1–C.3)** Multimodal imaging findings overlaid on MP2RAGE MRI. The secondary MRI morphometry (MAP) cluster (initially not verified by expert review and outlined in bright yellow) and hdEEG source imaging (ESI, sLORETA method, yellow-red) colocalize in the left mesial orbitofrontal cortex. **(D)** The three-dimensional electrode implantation scheme showing nine sEEG electrodes, with FBL (blue) and FIL (green) electrodes targeting the orbitofrontal cortex. Color-coded spheres represent different electrode trajectories and contact positions. **(E)** Stereoelectroencephalography recordings during seizure onset. Electrode contacts FBL1-FBL2 within the mesial orbitofrontal cortex show a characteristic seizure initiation pattern (red arrow) with initial gamma activity followed by repetitive spiking.

In our study, we frequently observed concordance between the visually verified MRI morphometry cluster and the hdEEG cluster showing the most frequent IEDs (Figure 3). However, it is important to note that LTM-ESI and MSI were performed in a much smaller subset of patients (Table 1). While multimodal imaging can sometimes yield entirely negative results, a second-look visual MRI analysis may reveal subtle structural abnormalities. These abnormalities, once identified, can be surgically removed following sEEG evaluation, leading to favorable postsurgical outcomes, as seen in one patient (P11). In another patient (P15), the MRI morphometry cluster did not uncover recognizable changes on visual MRI evaluation, and only the hdEEG cluster with the second-highest IED count was concordant with the seizure onset zone (SOZ) identified using sEEG (Figure 5).

## Discussion

### Main results

Multimodal imaging can effectively guide the planning of sEEG in patients with focal epilepsy, particularly when conventional MRI fails to reveal clear structural lesions. This includes patients who experience non-lesional epilepsy upon clinical review, those with only subtle structural abnormalities that require advanced imaging techniques for detection, and those with a history of prior epilepsy surgery. High-resolution non-invasive information enables careful testing of well-founded hypotheses regarding the potential epileptic focus using sEEG. sEEG not only delineates the SOZ for successful tailored epilepsy surgery in a significant number of patients but also helps rule out resective surgery in others. Multimodal concordance with the SOZ identified through sEEG is associated with favorable postsurgical outcomes. Another critical factor is that the electro-clinical hypothesis should be compatible with a single epileptogenic focus. In our study, seizure semiology consistently lateralized correctly in all but two patients (P8 and P15). In one of these two cases, invasive sEEG revealed a multifocal SOZ.

### Successful sEEG implantation and limited complications

The use of multimodal imaging enabled highly targeted implantation strategies with a limited number of sEEG electrodes. Our current approach, supported by dedicated planning software with 3D segmentation, allows for accurate placement of sEEG electrodes. Modern stereotactic planning systems, now widely adopted across epilepsy centers, offer significant advantages over earlier methods by providing greater flexibility in selecting trajectory orientations (37). The increased degrees of freedom in sEEG trajectories enable reaching multiple targets with a single electrode trajectory, thereby minimizing the number of electrodes required. These technological advancements collectively reduce the risk of complications, such as bleeding or infection (38–40).

### Success in the difficult patient group with prior surgery

MRI morphometry demonstrated the highest concordance rates with the SOZ identified by sEEG, making it particularly valuable for the planning of sEEG electrode implantation (Table 1). Even in patients with prior surgery, thorough multimodal presurgical diagnostics can still lead to seizure-free postsurgical outcomes. However, the overlap between the SOZ and language representations is a factor that, regardless of non-invasive multimodal concordance results, may negatively impact postsurgical outcomes, as seen in patient 14 (41, 42).

### Contribution of multimodal imaging

The presurgical evaluation of focal epilepsy benefits from the integration of multiple imaging modalities, with both structural and functional methods contributing additive information. Our findings demonstrate that MAP concordance with the sEEG-defined SOZ was enhanced when supported by additional functional imaging methods, particularly in identifying a monofocal SOZ. The value of multimodal imaging is highlighted by several key observations in our study and previous research.

MAP demonstrated important synergies with multiple functional imaging methods in our cohort. The upset plot reveals frequent concordance between MAP and other modalities, particularly with hdEEG-ESI (Figure 3), which was more commonly available in our study. This multimodal concordance aligns with previous studies showing that integration of structural and functional imaging methods improves the identification of the epileptogenic zone and prediction of surgical outcomes in non-lesional focal epilepsy (43, 44). While MSI was less frequently available in our cohort, the observed concordance between MAP and various functional imaging techniques suggests that combining structural information from MAP with complementary functional data provides a more comprehensive understanding of the epileptogenic network.

Functional imaging methods each offer distinct advantages in the presurgical workup. MSI and ESI provide high temporal resolution that can track the propagation of epileptiform activity. ESI, whether derived from hdEEG or LTM recordings, showed concordance with the SOZ at IED peaks in our cohort. However, the utility of these methods can be influenced by various factors. Short-duration hdEEG and MEG recordings (1–2 h) may miss less frequent IEDs (34), while longer LTM recordings can capture more representative IED patterns and provide better signal-to-noise ratios for analyzing propagation patterns (25, 27, 28).

The impact of prior surgical interventions on source imaging accuracy deserves particular attention in future methodological research. More accurate head models, such as finite element models (FEMs), may further increase the accuracy of ESI and MSI (45, 46). In patients with prior craniotomy and resection cavities, electrical conductivity likely deviates from normal tissue properties, and accurately accounting for these factors will probably further improve the accuracy of ESI and MSI.

It is important to note that non-concordant findings from functional imaging methods may still provide valuable information by delineating the irritative zone, which typically extends beyond the SOZ. This highlights the importance of interpreting each modality within the broader context of presurgical evaluation. Furthermore, while our results support the value of multimodal imaging, the limited availability of certain methods (particularly LTM-ESI and MSI) in many European centers due to a lack of specific reimbursement policies (47) restricted our ability to fully evaluate their complementary role. Larger studies are needed to further validate these findings and establish optimal protocols for integrating multiple imaging modalities in presurgical evaluation. New trials should also evaluate the cost-effectiveness of targeted sEEG implantation based on multimodal imaging (48). While the variable availability of modalities in our cohort reflects real-world clinical practice, it demonstrates the feasibility of integrating all available imaging methods to optimize sEEG planning.

### Clinical aspects

Our cohort highlights several important considerations for the clinical application of advanced imaging methods. A key challenge is the management of false positive findings across all imaging modalities. For MAP specifically, many false positive findings can be effectively ruled out through careful visual correlation with FLAIR or other T2-weighted MRI sequences. However, our experience with the patient P15 (Figure 5) demonstrates that secondary MAP clusters may sometimes contain the true SOZ, suggesting that sEEG electrode placement should consider both primary and secondary hypotheses in selected cases. The effective use of multimodal imaging requires a deep understanding of each modality’s strengths and limitations in specific clinical scenarios. For instance, MSI demonstrates particular utility in patients with a perisylvian SOZ and tangential sources (49–51).

Our experience also emphasizes that when multiple imaging modalities fail to identify clear abnormalities, meticulous visual evaluation remains crucial. This was exemplified by the patient P11, in whom subtle morphological abnormalities were only identified through careful second-look visual evaluation of the high-resolution MRI. These patients underscore the continuing importance of expert visual analysis alongside advanced imaging techniques.

### Limitations

There are several limitations that should be acknowledged. The retrospective design and small cohort size (n = 15) limit statistical power and generalizability of these findings, particularly for modalities used in fewer patients, such as ESI or MSI. One notable limitation is that, aside from MRI morphometry, no other ancillary diagnostic tests were performed uniformly across all patients, which may introduce selection bias and overstate the relative contributions of ESI or MSI. The low number of ESI and MSI findings, performed in only a subset of patients, further limits our ability to assess each method’s individual benefit and might overrepresent their clinical relevance (22). The observed concordance rates should be viewed as hypothesis-generating data that warrant prospective validation in larger, multi-center studies with uniform diagnostic testing and standardized multimodal protocols. However, this pilot study provides proof-of-concept evidence that such studies are warranted.

### Implications for clinical practice

Despite the limitations of our retrospective cohort study, in the presurgical evaluation of patients with focal epilepsy where conventional MRI shows no clear lesions, all available multimodal imaging methods—such as MRI morphometry, ESI based on hdEEG and LTM, and simultaneous ESI/MSI—may be considered for sEEG electrode placement. This approach also applies to patients with a history of prior epilepsy surgery (52). Since different multimodal imaging techniques highlight distinct aspects of the epileptogenic area, only a targeted combination of these methods may prove particularly helpful in generating well-substantiated hypotheses about the epileptic focus. These hypotheses can then be further validated using sEEG.

## Data Availability

The datasets presented in this article are not readily available because ethical restrictions. Requests to access the datasets should be directed to marcel.heers@uniklinik-freiburg.de.
